# Editorial

**Published:** 2010-05-01

**Authors:** Karim Vessal

**Affiliations:** *Academy of Medical Sciences of I.R. Iran*

In the field of transplantation, there is little hope that even near-modular organs will ever be available in the foreseeable future, or any major breakthrough to occur in xenotransplatation. The transplantation community is, and will remain dependent on the viability of the harvested organ to survive and secure further growth of this novel field. This depends much on the timing of harvesting in order not to incur avoidable failure. To achieve this, public trust in the medical handling the dying patient remains one of the prime objectives.

In the previous issue of this journal, Delmonico expounded on the definition of death before engaging in organ recruitment with the intent of securing medical and judicial legitimacy for a successful transplantation. With a concluding remark and a citation from the late Pope [1] on the issue of dichotomy of cardiac-respiratory versus neurological, he tends to favor the latter. Fry-Revere, in the current issue of the journal, however, inclines toward a definition based on circulation, because of inherent incertitude regarding determination of total brain death.

It should be noted that opinion expressed by Pope is apparently his own as a learned, pious individual since neither Judeo-Christianity nor Islam has set any strict criteria on the bodily signs of the separation of soul and onset of death. The mandates of the Abrahamic religions on death and dying relate more to the salvation of the soul of the dying person and decrees towards observing sanctity of the corpse. Overall, the decision on the time of death aside from the attending professionals is strongly subjective and mostly confirmed or decided by the clergy and senior relatives present.

Development of organ transplantation in Iran as well as other countries of the region started around one decade later than the United States. While the progress of the new field has been quite impressive, the experience acquired so far tends to be somewhat different compared to that accumulated in the West.

We believe that the public criteria regarding the onset of death are amazingly more lax than that set in medical profession. In this part of the world, the guidelines promulgated in local traditional medicine, has been of respiratory category with holding of mirror in front of the subject's mouth and watching for the presence of a blurred reflection through exhaled meanwhile checking for the absent radial pulse.

In transplantation medicine, the horizon, in this part of the world, appears more bright because of the public belief is still inclined to accept a noble and altruistic motivation in the relevant professionals. The public in the emotionally engaged state of bereavement is less worried about the intention of the operating team rather than salvation and maintaining the integrity of the body of the beloved one. 

We believe that cultural factors are the third entity to be considered while contemplating organ procurement. It is an undeniable fact that in an emerging scenario, organ donation does not rank high in the minds of the kin of a dying person, hence bringing closer these divergent ideas might help prevent many potential mistrusts.

We believe that along with due considerations being paid to the prevailing culture and level of education, the relatives should informed of the outcome without any recourse made to the judicial side of the matter and relegate pronouncement of death to their arbitration and confirmation.
